# ER Stress and the Unfolded Protein Response: Homeostatic Regulation Coordinate Plant Survival and Growth

**DOI:** 10.3390/plants11233197

**Published:** 2022-11-22

**Authors:** June-Sik Kim, Keiichi Mochida, Kazuo Shinozaki

**Affiliations:** 1 RIKEN Center for Sustainable Resource Science, Yokohama 230-0045, Japan; 2 Institute of Plant Science and Resources, Okayama University, Kurashiki 710-0046, Japan; 3 Microalgae Production Control Technology Laboratory, RIKEN Baton Zone Program, Yokohama 230-0045, Japan; 4 School of Information and Data Sciences, Nagasaki University, Nagasaki 852-8521, Japan; 5 Graduate School of Nanobioscience, Yokohama City University, Yokohama 236-0027, Japan

**Keywords:** endoplasmic reticulum (ER), ER stress, unfolded protein response (UPR), gene regulation, stress response, defense, vegetative growth, reproduction

## Abstract

The endoplasmic reticulum (ER), a eukaryotic organelle, is the major site of protein biosynthesis. The disturbance of ER function by biotic or abiotic stress triggers the accumulation of misfolded or unfolded proteins in the ER. The unfolded protein response (UPR) is the best-studied ER stress response. This transcriptional regulatory system senses ER stress, activates downstream genes that function to mitigate stress, and restores homeostasis. In addition to its conventional role in stress responses, recent reports indicate that the UPR is involved in plant growth and development. In this review, we summarize the current knowledge of ER stress sensing and the activation and downstream regulation of the UPR. We also describe how the UPR modulates both plant growth and stress tolerance by maintaining ER homeostasis. Lastly, we propose that the UPR is a major component of the machinery that balances the trade-off between plant growth and survival in a dynamic environment.

## 1. Introduction

The endoplasmic reticulum (ER) is a eukaryotic organelle that serves as a major assembly site for protein superstructures [1]. At least one-third of cellular proteins are processed at the ER and directed toward secretory pathways, where they are folded into their functional forms [2]. Various stressful stimuli trigger fluctuations in the cellular environment that cause misfolded and unfolded proteins to accumulate inside the ER, resulting in ER stress.

The ER quality control (ERQC) system monitors and fortifies ER function to maintain homeostasis [3,4,5]. In this system, ER-resident proteins such as chaperones, cochaperones, lectins, redox enzymes, and glucosidases have designated roles in protein processing. The ER-associated protein degradation (ERAD) pathway reduces the unfolded-protein load through a ubiquitin-dependent proteasomal protein catabolic process. The overaccumulation of unfolded proteins in the ER triggers gene regulatory machinery that enforces ER function by promoting the expression of ERQC components, namely, the unfolded protein response (UPR).

As ER stress is induced by a broad spectrum of stress categories and hormonal responses, the UPR influences diverse biological processes, from stress tolerance to growth and development. In this review, we discuss the current understanding of plant ER stress, with a focus on UPR triggers and downstream regulation in the model species Arabidopsis (*Arabidopsis thaliana*). We also provide an updated overview of the redundant and unique biological roles of two main UPR pathways.

## 2. Two Distinct Plant UPR Pathways

**Figure 1:**
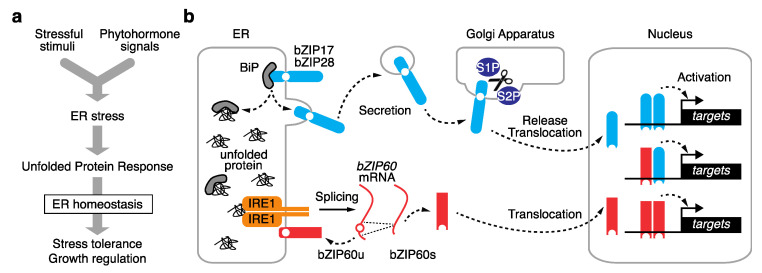
**Schematic view of the plant ER stress signaling pathway.** (**a**) The plant ER stress response transduces both exogenous stress stimuli and endogenous phytohormone signals into the unfolded protein response (UPR) to govern stress tolerance and growth regulation. (**b**) Two eukaryote-wide UPR pathways are activated by distinct pathways. Arabidopsis bZIP17 and bZIP28 are activated by post-translational proteolysis at the Golgi apparatus, and bZIP60 is activated by alternative splicing. The activated bZIPs are translocated to the nucleus, where they activate downstream target genes.

Eukaryotes have developed multiple UPR pathways that communicate with the nucleus and vary by evolutionary lineage [6]. In vascular plants, two eukaryote-wide pathways have been identified with some plant-specific features. Each UPR pathway consists of unique components, including stress sensors, activators, and transcription factors. A dedicated subclade of basic leucine zipper (bZIP)-type transcription factors is involved in the UPR, of which bZIP17, bZIP28, and bZIP60 have been identified in Arabidopsis. These bZIPs are localized to the ER membrane, where two distinct machineries activate them for translocation into the nucleus (Figure 1).

### 2.1. Proteolysis-Dependent UPR Pathway

Arabidopsis bZIP17 and bZIP28 are counterparts of mammalian activating transcription factor 6 (ATF6) and mediate a UPR pathway via post-translational proteolysis [5]. In each of these transcription factors, the N-terminus containing the bZIP domain faces the cytosol and is connected by a transmembrane domain to the C-terminal tail, which is exposed to the ER lumen [6,7]. In response to increasing ER stress levels, these two bZIPs are secreted into the Golgi apparatus, where proteases SITE-1 PROTEASE (S1P) and S2P cleave the proteins between the bZIP and transmembrane domains. The released N-terminus possessing the bZIP domain is freely translocated into the nucleus and activates downstream genes involved in the UPR [6,7] (Figure 1b).

In a paralogous manner to that observed in human ATF6, the lumen-facing region of Arabidopsis bZIP28 functions as a stress sensor [8]. During ER homeostasis, ER-resident chaperone BINDING PROTEIN 3 (BiP3) binds to the lumen-facing region of bZIP28, preventing its activation. When ER stress increases, BiP3 preferentially binds to unfolded proteins, allowing bZIP28 to be secreted into the Golgi apparatus, where it is activated (Figure 1b). Further details have not been fully elucidated, but recent studies identified a key ER-resident chaperone and a novel chemical affecting ATF6 trafficking in mammals [9,10]. This could provide insight into the underlying mechanism in plants.

### 2.2. mRNA Splicing-Dependent UPR Pathway

The other UPR pathway is mediated by Arabidopsis bZIP60, which is activated by alternative splicing [11,12,13]. bZIP60 counterparts exist in most eukaryotes, from budding yeast to mammals, and are known as X-box binding protein 1 (XBP1) [6]. In the absence of activation by ER stress, the major isoform of *bZIP60* mRNA encodes a protein with a transmembrane domain that anchors it to the ER membrane, thus compromising its transcription factor activity. When ER stress is elevated, stress sensor and activator INOSITOL-REQUIRING ENZYME 1 (IRE1) in the ER lumen specifically splices the *bZIP60* mRNA to remove its transmembrane domain, allowing for bZIP60 to migrate into the nucleus to activate the UPR (Figure 1b). Two major IRE1 homologs (IRE1a and IRE1b) were extensively studied in Arabidopsis [13,14,15,16]. Both IRE1s are transmembrane proteins that localize to the ER membrane, where their ER lumen-facing domain functions as a stress sensor, and their cytosol-facing domain splices or degrades target mRNAs. These two homologs are functionally redundant during bZIP60 activation in response to ER stress. A third IRE1 homolog in Arabidopsis was recently characterized. Unlike IRE1a and IRE1b, IRE1c lacks the lumen-facing domain that functions as the ER stress sensor; however, biochemical and genetic experiments revealed that IRE1c couples with IRE1b to play critical roles in gametogenesis [17,18]. *Arabidopsis lyrata*, a close relative of *Arabidopsis thaliana*, contains a fourth IRE1c-like homolog whose role in the UPR is unclear [17]. By contrast, only a single *IRE1* copy has been identified in rice (*Oryza sativa* subsp. *japonica*), and the knockout of this gene is lethal [19].

In addition to bZIPs, three NAC (NAM, ATAC, and CUC)-type transcription factors (NAC062, NAC089, and NAC103) play regulatory roles in the plant UPR [20,21,22]. The expression of these genes is induced by ER stress, and their expression is diminished in *bzip60* single mutants [20,22,23], suggesting that these NACs may function as subordinates of the IRE1-bZIP60 pathway. However, the translocation mechanism of these NACs from the ER to the nucleus remains uncertain; novel plant-specific pathways are expected to be discovered upon further investigation.

### 2.3. Modes of Action of UPR bZIP Dimers

bZIP transcription factors form dimers that bind to their target DNA sequences [24]. These transcription factors can form homo- and heterodimers even across subclade boundaries. Different dimers are thought to have different target preferences that could result in diverse downstream gene regulatory pathways. The three UPR bZIPs (bZIP17, bZIP28, and bZIP60) also form both homo- and heterodimers in every possible combination [23,25]; their different modes of action have been analyzed using combinations of their knockout mutants. 

In Arabidopsis, bZIP28 and bZIP60 are commonly accepted as the foremost combination governing canonical UPR downstream regulation [7,26]. The expression of most known ERQC component genes was impaired by the double mutation of *bZIP28* and *bZIP60* (*bzip2860*). A significant number of genes, including *BiP3*, *CNX1* (*CALNEXIN 1*), and *ERO1* (*ER OXIDOREDUCTIN 1*), also fully lost their stress-inducible expression in the double mutant [23]. Moreover, these two bZIPs have their own specific targets. A single *bzip28* mutation was sufficient to inhibit the stress-responsive expression of *CRT2* (*CALRETICULIN 2*), *SHD* (*SHEPHERD*), and *SDF2* (*STOMATAL-DERIVED FACTOR2*), whereas the single *bzip60* mutation abolished the stress-responsive induction of *PDI9* (*PROTEIN DISULFIDE ISOMERASE 9*), *SAR1A* (*SECRETION-ASSOCIATED RAS 1A*), and *SEC31A* (*SECRETORY 31A*) [23,25,27]. Consistently, *bzip2860* plants showed reduced tolerance to chemical-induced ER stress, and the *bzip28* and *bzip60* single mutants had a similar, but weaker, phenotype [28].

bZIP17, a homolog of bZIP28, is also a transcriptional activator that responds to ER stress [29], although its role in the canonical UPR appears to be auxiliary to that of other UPR bZIPs. Under ER stress conditions, transcriptomic changes in vegetative tissues caused by the double mutation of *bZIP17* and *bZIP60* (*bzip1760*) are only slightly different from those caused by the single *bzip60* mutation, and the single *bzip17* mutation also resulted in only subtle changes [23]. Recent studies revealed that bZIP17 plays important roles in inflorescence tissues [30,31,32], with specific target genes including *HOMEOBOX 7 (HB-7*) and *DELTA1-PYRROLINE-5-CARBOXYLATE SYNTHASE 1 (P5CS1*) [32,33].

## 3. UPR Action in Stress Responses 

**Figure 2:**
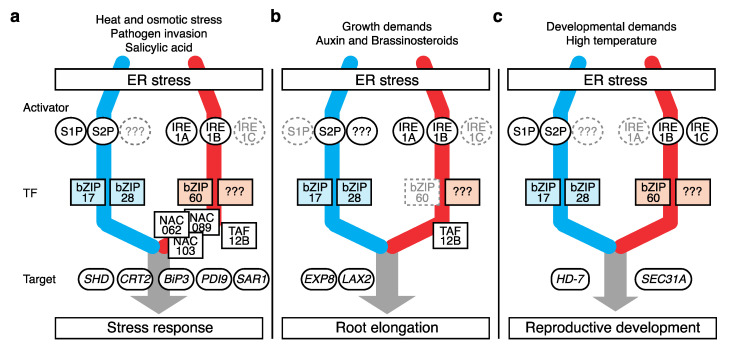
**Different modes of plant UPR action.** Plant UPR components governing biological processes including (**a**) canonical stress response, (**b**) root elongation, and (**c**) reproductive development. Components with an auxiliary role are marked in gray. Suspected but unknown components are marked as “???”. Only representative targets are shown. The color scheme for the two UPR pathways is the same as that in Figure 1.

While monitoring ER homeostasis, the UPR responds to a broad range of signals, including stress stimuli, phytohormonal reactions, and developmental phase changes, which interfere with protein folding. By enforcing ERQC, the UPR imparts tolerance toward these stressors in plants (Figure 2a and Table 1).

Heat stress is one of the best-studied UPR triggers due to its striking effect on protein structure. Single mutants *bzip17*, *bzip28*, and *s2p* have reduced heat tolerance [32,34,40]. The double mutants of *bZIP28* and *bZIP60* (*bzip2860*), and *IRE1a* and *b* (*ire1ab*) are especially vulnerable to high temperatures during reproduction [30,36]. Furthermore, both proteolysis-dependent bZIP28 and splicing-dependent bZIP60 activation can be induced by heat-shock treatment [12,34]. 

Osmotic stress is another UPR trigger in plants. High-salinity and high-osmolarity conditions trigger the activation of bZIP17 and its translocation to the nucleus [31,39], and the ectopic expression of *bZIP17* improved plant resilience to salt stress [29]. Salt-stress-induced bZIP17 proteolysis was diminished in the single *s1p* mutant [39], and the single *s2p* mutant also exhibited reduced tolerance to desiccation [40] and reduced sensitivity to abscisic acid (ABA), the plant hormone that mediates drought stress signaling [33].

The UPR also plays roles in biotic stress responses (Table 1). Multiple genetic studies have provided evidence supporting the pivotal role of the IRE1-bZIP60 UPR pathway in plant defense against various pathogens and viral infections. The *ire1a*, *ire1b*, and *bzip60* mutants showed weakened defense against inoculation with *Plantago asiatica mosaic virus*, *Turnip mosaic virus* [37,38], and the bacterial pathogen *Pseudomonas syringae* [41]. *bZIP60* mRNA splicing and activated protein migration to the nucleus were also observed during viral infection [37,38]. The expression of UPR-bZIP genes *bZIP60*, *bZIP17*, and *bZIP28* is induced upon viral infection, although the extent of induction varies among viruses [38].

Salicylic acid (SA) is a well-studied plant hormone involved in defense signaling [45,46]. According to current models, the SA-responsive transcription factor NON-EXPRESSOR OF PR GENES 1 (NPR1) plays a central role as an SA receptor and transcriptional activator of defense genes in response to infection. The ectopic expression of *NPR1* induced the expression of ER chaperone genes [47]; however, another study showed that both Arabidopsis UPR pathways are activated by exogenous SA application via an NPR1-independent pathway [48]. Furthermore, ER stress induces SA biosynthesis in rice [49]. Together, these observations suggest that plant ER stress signaling and SA-mediated defense signaling are interconnected via crosstalk-mediated mechanisms, which may help in finetuning a multilevel defense response against various infections.

## 4. Role of the UPR in Root Growth

In Arabidopsis, mutations within UPR components often result in defects in primary root growth even under unstressed conditions (Figure 2b and Table 1). Among the resulting mutants, the double mutants of *bZIP17* and *bZIP28* (*bzip1728*) showed the most severe phenotype, exhibiting a more than 90% reduction in vertical root growth compared to the wild type [23]. No defect was observed in *bzip17* or *bzip28* single mutants, indicating that these bZIPs redundantly govern basal root elongation [16,23]. The aboveground shoot growth of *bzip1728* was also severely repressed [23]. The root–shoot grafting of *bz1728* shoots with wild-type roots substantially recovered the shoot growth of this mutant, whereas *bz1728* root growth was not improved by grafting to wild-type shoots [35]. Thus, inhibited root growth is the major defect caused by the *bz1728* mutation.

Defective root growth was also observed in the *s2p* mutant, which lacks S2P, the known UPR activator of bZIP17 and bZIP28 [40,50]. The root growth defect in the *s2p* mutant is mild compared to that in *bzip1728* [23], suggesting that other, unidentified UPR activators exist for the two bZIPs (Figure 2b). S1P is another known activator associated with bZIP17 activation, although its mutation did not alter root growth, and the double mutation of *S1P* and *S2P* (*s1p2p*) resulted in the same root growth phenotype as that of the *s2p* single mutant [23,39]. Thus, S1P plays a limited role in UPR-associated root growth, which might be due to the reported irrelevance of S1P activity for bZIP28 activation [51]. 

The other IRE1-bZIP60 pathway also participates in UPR-associated root growth. The double mutation of *IRE1a* and *IRE1b* (*ire1ab*) results in a significant reduction in vertical root growth [16,43,44]. Single mutants of these genes did not show such growth defects, indicating that the two IRE1s redundantly contribute to UPR-associated root growth. The mutants of IRE1-target *bZIP60* showed no defects in root growth [16,28,52]. Accordingly, the unknown targets of IRE1a and IRE1b are thought to be involved in UPR-associated root growth (Figure 2b). It is unclear whether the role of IRE1 in root growth depends on the UPR. An additional *bzip17* mutation incrementally worsened the root growth defect of *ire1ab* [44], and the double mutation of *ire1b* and *bzip28* also inhibited root growth [16]. Therefore, IRE1 appears to influence root growth through the UPR and its unknown targets, and is regulated in a coordinated manner with the proteolysis-dependent pathway.

The genetic basis of UPR-associated root growth remains to be elucidated. A recent forward genetic study attempted to revert the root growth defect of *bzip1728* and showed that vertical root growth in the established suppressor mutant *nobiro6* was recovered to 40% that of the wild type due to a malfunctioning of the general transcription factor component TAF12B (TBP-ASSOCIATING FACTOR 12B) [35]. In the *bzip1728* mutant, the expression of hundreds of genes was either upregulated or downregulated. The upregulated genes are enriched in stress-responsive genes, including *bZIP60* and its known downstream genes, whereas a substantial proportion of the downregulated genes are growth-promoting genes [23]. In *nobiro6*, the addition of the *taf12b* mutation dramatically attenuated the expression of the genes upregulated in *bzip1728*, whereas the expression of the downregulated genes was mostly sustained [35]. This indicates that the root growth defect of *bzip1728* is mainly due to excessive UPR activation (Figure 2b). 

The signaling of phytohormones auxin [53] and brassinosteroids (BRs) [40] might also be associated with UPR-mediated root growth regulation. Che et al. (2010) reported that BR signaling to the root occurs through the UPR, as the BR-responsive root growth defect was significantly alleviated in the *s2p* mutant. UPR coregulator TAF12B also functions in plant responses to phytohormones such as ethylene [54] and cytokinin [55]. A recent study indicated that cell-wall growth attenuator THESEUS1 reduces ER stress-induced root growth in association with ABA signaling [56]. Collectively, the plant UPR appears to integrate a broad range of external stimuli and plant hormonal signals that both positively and negatively modulate root growth. 

## 5. Role of the UPR in Reproductive Development

Plant UPR machineries play critical roles during the reproductive stages of development. The mutation of genes encoding several UPR components cause defects in plant fertility (Figure 2c and Table 1). The *ire1ab* mutant is characterized by abnormal pollen development under high-temperature conditions, which was ameliorated by the ectopic expression of *SEC31A*, a downstream target of bZIP60 [36]. Additionally, the *bZIP60* promoter is highly activated in inflorescence tissues, and *bZIP60* mRNA is spontaneously spliced to encode the active protein [27,36]. The single mutation of the plant-specific isoform *IRE1C* (*ire1c*) resulted in no significant defects in vegetative or reproductive development, but the double mutation with *IRE1B* (*ire1bc*) disrupted gametogenesis, and the triple *IRE1* mutation (*ire1abc*) is likely lethal [17,18]. Notably, the homozygote of a T-DNA mutant allele of *IRE1B* (SALK_018150) is nonviable, but the underlying mechanism is unknown [15,42]. As part of the proteolysis-dependent UPR pathway, bZIP17 also plays a role in plant reproduction under stress. A single mutation of *bzip17* resulted in greater reductions in fertility and silique growth under heat stress than those observed in the wild type [32], and the seeds of these mutants showed reduced drought tolerance [31].

The coordinate regulation of the two UPR pathways was also observed (Table 1). A triple mutant of *bZIP28* and two full-length *IRE1s* (*bzip28ire1ab*) was lethal, and the viable heterozygous plant *bzip28^+/^*^−^*ire1ab* showed severely reduced pollen viability [16]. A triple mutant of three UPR bZIPs (*bzip172860*) was also likely not viable, and the viable heterozygous line *bzip176028^+/^*^−^ was infertile, with abnormal development of inflorescence tissues [23]. BiP chaperones also function in both male and female gametogenesis [57,58]. In rice, *BiP* paralogs have been targeted in biotechnological studies to improve grain quality. Enhancing *BiP* expression by promoting the accumulation of the bZIP60-paralog OsbZIP50 increased seed storage protein content [59], and a recent study demonstrated that grain chalkiness is controlled by regulating the activity of the bZIP28-paralog OsbZIP60 [60]. Collectively, these observations indicate that the UPR promotes reproductive development in plants and thus has potential agronomic applications.

## 6. Questions and Perspectives

Although there have been tremendous efforts to elucidate the genetic and physiological roles of the plant UPR and its underlying mechanisms, questions still remain. *bZIP60* expression is self-inducible: the activated bZIP60 protein targets the *bZIP60* promoter to induce its expression [11]. However, the mechanism by which the other two bZIP genes are induced by stress is unclear. An understanding of this process would expand our knowledge of the plant UPR and post-transcriptional activation machineries in general, and potentially reveal novel promoter structures involved in stress responses. 

The downstream regulation of the UPR also warrants further study. The constitutive activation of bZIP17 or bZIP28 frequently causes severe growth repression in plants, but this was not observed for bZIP60 [27,29]. Therefore, these three bZIPs participate in at least two UPR pathways and have different target preferences. Considering our knowledge of coregulating factors such as NF-Y [25], HY5 [61], TAF12b [35], GBF2 [62], and the COMPASS-like complex [63], the further exploration of the downstream regulation of the UPR would clarify how the plant UPR governs both stress tolerance and growth modulation via crosstalk with other stress and phytohormone-responsive regulatory pathways (Figure 2). 

While the current article was under review, another review article on the plant UPR was published independently [64]. This review describes how the plant UPR responds to a broad spectrum of stress signals and regulates several biological processes, including cellular homeostasis, vegetative growth, and reproductive development. Future studies on the genetic and biochemical bases of stress sensing and on the activation and downstream regulation of the UPR could reveal the major role of the plant UPR in the trade-off between plant growth and survival in the wild.

## Figures and Tables

**Table 1:** Physiological defects and characteristics of knockout mutants of UPR components in Arabidopsis (*A. thaliana*).

Knockout Mutant	Viability	Stress Tolerance	Primary Root Growth (% of Wild Type)	Other Characteristics
*bzip17*	n.s.	Reduced tolerance to heat [32] and salinity [31]	100% [16,23]	Ectopic expression enhanced salinity tolerance [29]
*bzip28*	n.s.	Reduced tolerance to heat [34]	100% [16,23]	-
*bzip1728*	n.s.	-	10% [23,35]	Shoot growth defect recovered by grafting to wild-type roots [35]
*bzip60*	n.s.	Reduced tolerance to heat [36], viral infection [37]	100% [16]	-
*bzip1760*	n.s.	Reduced tolerance to viral infection [38]	100% [16,23]	-
*bzip2860*	n.s.	Reduced tolerance to heat [30]	100% [16,23]	-
*bzip172860*	Lethal [23]	n.a.	n.a.	-
*s1p*	n.s.	Reduced tolerance to salinity [39]	100% [39]	-
*s2p*	n.s.	Reduced tolerance to heat [36], drought [40]	40% [40]	-
*s1p2p*	n.s.	n.a.	40% [23]	-
*ire1a*	n.s.	Reduced tolerance to pathogen [41]	100% [16]	-
*ire1b*	One lethal mutant [15,42]	n.s.	100% [16]	-
*ire1c*	n.s.	n.a.	100% [18]	-
*ire1ab*	n.s.	Reduced tolerance to heat [36], viral infection [37]	60% [43]	-
*ire1bc*	Decreased pollen viability [17]	n.s.	100% [17]	-
*ire1abc*	Lethal [17,18]	n.a.	n.a.	-
*bzip17ire1a*	n.s.	n.s.	100% [44]	-
*bzip17ire1b*	n.s.	n.a.	60% [44]	-
*bzip17ire1ab*	n.s.	n.a.	10% [44]	Delayed flowering [44]
*bzip28ire1ab*	Lethal [16]	n.a.	n.a.	-
*bzip60ire1ab*	n.s.	n.a.	60% [16]	Similar genetic defects to *ire1ab*

n.s., no significant difference from the wild type. n.a., not applicable.

## Data Availability

Not applicable.
